# Priorities of continuing training for global practitioners in Kashan University of Medical Sciences 2013

**Published:** 2015

**Authors:** M Aghajani, F Jahangir, M Saiedi Majd, N Jahangir

**Affiliations:** * Department of Psychiatric Nursing, Trauma Nursing Research Center, Kashan University of Medical Sciences, Kashan, IR Iran,; ** Kashan University of Medical Sciences, Kashan, IR Iran

**Keywords:** continuing education, medical sciences, general practitioners

## Abstract

**Introduction:** Continuing the medical education has already been a concern, as a universal principle and necessity in the universe, so that the World Health Organization (WHO) has specified it as an urgent necessity. This research objective was the assay of priorities of continuing training for global practitioners at Kashan University of Medical Sciences.

**Methods and Materials:** In this cross-sectional study, 212 out of 600 general practitioners take parting in the keeping on the medical education programmes held by KUMS were covered. After being interviewed about their needs and interest in the programmers, a survey including demographic information such as the education needed, way of teaching, etc., was given to them. The data were then analyzed by SPSS v 11.5.

**Results:** 137 (64.6%) cases were males and 75 (48.3%) were women. Also, 38 (17.9%) individuals were working in emergency wards. Findings indicated that the first priority of the programs was the internal diseases emergency, and then the pediatric common diseases. The third priority was the infectious common diseases.

**Conclusions:** The needs of the CME and the use of the appropriate methods to match the content of the training needed the assessment of the GP training program design painting by the learners, thus enhancing quality and the efficiency of CME applications.

## Introduction

Continuing medical education has already been a concern, as a universal principle and necessity in the world, so that the WHO has also recognized it as an urgent necessity. At the world conference of medical education in Edinburgh 1993, CME was emphasized as a necessary activity for the maintenance of professional standards and skills. It was declared that in order for the medical educations to be more effective and relevant, the educations should be given according to the needs of the graduates [**1**,**2**]. Since continuing medical education is to enhance the physicians’ knowledge and functionality, gain advantageous standards for the medical services matched with the needs of society, and promote the health level, the assessment and consideration of the needs of this group is of value. Various studies have been conducted on CME in different countries in which different methods for the assessment and design of the CME programmes have been evaluated. Davis investigated about 1% of the studies done in this regard. He declared that programmes provided for physicians or those addressed by the CME were all satisfactory and designed based on their needs, beliefs, and opinions. He hoped the programmes were more attractive and could promote professional capacities of the participants [**3**]. Also in Iran, continuing the medical education was given much attention. It is growing both from the quantitative and from the qualitative view point. Nevertheless, since various programmes were scheduled and performed for general practitioners, it is critical to what extent these programmes were principally designed and based on the requirements of learners or how they contributed to the promotion of providing health and therapeutic services. Few studies in Iran revealed that a part of CME programmes could not meet the real needs and concerns of the society due to an incorrect recognition and prioritization of educational needs. Therefore, the education given had a trivial value. For instance, according to a study conducted in the Medical University of Zahedan on the attitudes of general practitioners on the contents of CME programmes, 87.8% of the participants asked for more applied programmes, 78.2% were interested in previous discussions, information, and sources; 76.3% requested seasonal and domestic discussions selected according to surveys. Regarding the proportion of time to the contents of internal and surgical programmes, 85.3% and 83.9% of the physicians found it inappropriate, respectively. Half of the cases mentioned no positive point for the programmes. According to the previously said outcomes, monitoring factors and cases such as carefully selected discussions, setting times for each discussion, prioritizing domestic and seasonal diseases, using survey results for the selection of discussions, and giving information to participants about the contents and resources were recommended to modify continuing medical education [**4**]. Another study entitled “Attitudes of general practitioners towards their profession” was carried out in Medical Sciences Kashan University in 2001. The findings are as:

Mostly, physicians had a problem with the diagnosis and the interpretation of radiographs. They rarely had a problem with the examination of the cases. In the case of drug prescription, drug interactions were their main concern. General practitioners requested to perform CME courses in the field of emergency skills, nutrition, and radiography interpretation. Emergency skills, toxicities, and cardiac pulmonary arrest were the main problems physicians were encountered with. Consequently, meeting the physicians’ needs, especially those focused on by the physicians themselves was necessary to achieve the best outcomes to continue medical education. Therefore, since reviewing opinions of learners is considered one of the vital ways to enhance the continuing education quality, using their ideas could be effective and could guarantee the quality of the programmes. Thus, this study’s aim was to evaluate the attitudes of general physicians towards their educational priorities and programmes of the medical continuing education performed in Medical Sciences Kashan University during 2012 and 2013. 

## Methods

212 out of 600 general practitioners participating in the continuing medical education programmes held by Kashan University of Medical Sciences were considered in this research. After being interviewed regarding their needs and interest in the programmes, a survey including demographic data such as education needed, way of teaching, etc., was given to them. The information are then analyzed by SPSS. The validity of the study was assessed by studying at least 30 of the physicians by utilizing coefficient of Cronbach’s alpha. Related experts confirmed the accuracy of the research according to similar studies.

## Results

This study covered 212 general practitioners and experts (10 cases) in Kashan of whom, 137 (64.6%) were mens and 75 (48.3%) were females. 95 (45%) were recruited officially or by contract, 1 (5%) was working on a project, 66 (31.1%) were businessmen, and 10 (4.7%) were faculty members (**Table 1** and **2**).

**Table 1:** Prevalence distribution of priority

Methods of Teaching in CME	Times of being selected as the first priority (%)	n (%)
Methods of Lecturing in CME	64 (30.3)	212 (100)
Methods of questioning and Answering in CME	74 (22.2)	212 (100)
Methods of Group Work in CME	35 (16.5)	121 (100)
Methods of teaching Case Reports in CME	71 (33.5)	212 (100)

**Table 2:** Prevalence distribution of priority for continuing medical education in the view of general practitioners

Type of continuing the education programme	Times of being selected as the first priority (%)	n (%)
Continuing education programmes	76 (35.8)	212 (100)
Conferences of CME	9 (4.2)	212 (100)
Seminars of CME	8 (3.8)	212 (100)
Workshops of CME	13 (6.1)	212 (100)
Educational symposium of CME	6 (2.8)	212 (100)
Congresses of CME	18 (8.5)	212 (100)
Professional short-term programmes of CME	29 (13.7)	212 (100)
Self-educated programmed in CME	37 (17.5)	212 (100)

38 (17.9%) individuals are doing in emergencies, 41 (19.3%) in hospitals, 62 (29.2%) in private offices, 8 (3.8%) in private clinics, 18 (8.5%) in health and hygiene centers, and 14 (6.6%) in other centres. Among the 212 participants, 27 and 77 individuals were the youngest and oldest physicians, respectively with an average 41 years. 162 cases had a job test of 11.3 years (1-41 years of work experience) (**Table 3** and **4**).

**Table 3:** Prevalence distribution of priority for time of keeping on medical trading in the aspect of global practitioners

Day	Times of being selected as the first priority (%)	n (%)
Saturday	39 (18.4)	212 (100)
Sunday	13 (6.1)	212 (100)
Monday	20 (9.4)	212 (100)
Thursday	22 (10.4)	212 (100)
Wednesday	26 (12.3)	212 (100)
Tuesday	85 (40.1)	212 (100)

**Table 4:** Prevalence distribution of priority for continuing medical education in the view of general practitioners

Type of continuing the education programme	Times of being selected as the first priority (%)	n (%)
Internal diseases emergency	141 (66.5)	212 (100)
Pediatric common diseases	100 (47.2)	212 (100)
Infectious common diseases	100 (47.2)	212 (100)
Cardiovascular common diseases and emergency	98 (46.2)	212 (100)
Digestive common diseases	92 (43.4)	212 (100)

## Discussion 

The main purpose for the performance of the continuing education programmes for the general physicians or other groups addressed was to fulfill their basic needs so as to promote their professional abilities. Performing an appropriate assessment was the main way to achieve that goal. That helped the executers design more effective and useful programmes. The main priorities in this study included 141 (66%) cases of internal diseases emergency, 100 (47.2%) cases of pediatric common diseases, 98 (46.2%) cases of common cardiovascular diseases, 98 (46.2%) cases of cardiovascular disease emergency, 92 (43.3%) cases of digestive common diseases and paraclinic laboratorial results, 85 (40.1%), cases of pediatric diseases emergency, and 82 (38.7%) cases of surgical disease emergency. In their research “Continuing education needs of general practitioners”, Abolghasem Amini et al. obtained that their advantages are the following: injuries and events emergency (61.7%), heart internal diseases (60.4%), skin diseases (58.5%), internal common emergency (58.2%). As it was shown, heart diseases along with internal emergency had the highest rate of consistency in our study. Also, in a study conducted by Abbasalat Borji in Zahedan Medical University [**3**], internal diseases (85.9) were prioritized over others. That result was in consistency with the one in the present study. Abdollhossein Shokournia et al. believed that the main educational needs were related to the internal diseases just as mentioned in our study [**5**]. 

Moreover, that study showed that the physical educational activity in performing the continuing education programmes (52 cases, 24.5%), case report representation (71 cases, 33.5%), and type of programme (76 cases, 35.8%) were the main priorities. However, based on a study done in Zanjan in 1389, the common practitioners determined the continuing education programmes efficacy as average or low, which was not the same as that in our study [**6**]. The results gained by Mehdi Amirnia et al. [**7**] confirmed the inefficiency of the way of lecturing versus the active methods of teaching. According to a Canadian study, lecturing did not have any effect on changing the clinical function or health care, which was stated in our study as well. Programmes of the continuing education mostly focused on giving new and latest information about treating patients (92 cases, 43.4%). This was in agreement with the findings obtained by Mehdi Amirnia and colleagues. Mandana Shirazi et al. [**8**]. concluded that lecturing using videos (52%) and types of programmes (58.3%) are the most usual ways for continuing education, which was in line with our study. Also, this research indicated that the most suitable time to hold the programmes was on Thursdays (85 cases, 40.1%). The length of one-day programmes (84 cases, 39.6%) was two hours (43 cases, 20.3%), while for the programmes lasting for more than two days, the participants included 48 individuals (7.30%). These results were the same as those given by Abolghasem Amini et al. [**9**]. in the Medical University of Tabriz, in which the best days were Thursday (61%) and Friday (52%). The best time was before noon as well. According to our study, the top priorities in continuing education programmes included internal diseases emergency, common pediatrics diseases, infectious diseases, common cardiovascular diseases and emergency, and common digestive diseases, respectively.

## Results

Since the educational needs of general practitioners, the application of novel methods for the evaluation of the target population, and the consistency of educational content with the general practitioners’ educational needs all contribute to the promotion of continuing the education programmes, they will consequently lead to a general satisfaction among the learners. The top priorities in this study included internal diseases emergency, common pediatric and infectious diseases, common cardiovascular diseases, and common digestive problems, respectively.

**Acknowledgements**

The researchers would like to express their gratitude to the personnel of KUMS/ Iran.

**Financial Disclosure**

The authors says that they have not any competing interests.

**Funding/Support**

This study was funded and supported by the Deputy of Research, Kashan University of Medical Sciences (KAUMS).
